# Microstructure, Corrosion and Mechanical Properties of TiC Particles/Al-5Mg Composite Fillers for Tungsten Arc Welding of 5083 Aluminum Alloy

**DOI:** 10.3390/ma12183029

**Published:** 2019-09-18

**Authors:** Qibo Huang, Rouyue He, Chunxia Wang, Xin Tang

**Affiliations:** 1 Key Laboratory of New Processing Technology for Nonferrous Metal & Materials, Ministry of Education, Guilin University of Technology, Guilin 541004, China; qibo.huang@outlook.com (Q.H.); rouyue.he@hotmail.com (R.H.); 2 College of Material Science and Engineering, Guilin University of Technology, Guilin 541004, China

**Keywords:** TiC particles, TIG welding, mechanical properties, corrosion, first principle calculation

## Abstract

A semi-solid stir casting mixed multi-pass rolling process was successfully employed to manufacture TiCp/Al-5Mg composite filler wires with different contents of TiC particles. The 5083-H116 aluminum alloys were joined by tungsten inert gas (TIG) using TiCp/Al-5Mg composite weld wires. The microstructure, mechanical properties, fractography and corrosion behavior of the welds were evaluated. The results revealed that TiC particles were distributed in the welds uniformly and effectively refined the primary α-Al grains. The hardness and tensile strength of the welds were improved by increasing the TiC particle content, which could be attributed to the homogeneous distribution of TiC particles and the microstructure in the weld joints. Potentiodynamic polarization testing revealed that the corrosion resistance of the welds also increased with the addition of TiC particle contents. In addition, the stress corrosion cracking (SCC) susceptibility of the welds decreased as micro-TiC particles were introduced into the welds. The electronic structure of the Al/TiC interface was investigated by first principle calculation. The calculation showed that valence electrons tended to be localized in the region of the TiC-Al interface, corresponding to an addition of the overall work function, which hinders the participation of electrons in the composite in corrosion reactions.

## 1. Introduction

Aluminum and its alloys have been widely applied in aircraft, marine, aerospace and automotive industries because of their extraordinary properties, for example, light weight, high specific strength, excellent processing properties and corrosion resistance [1]. Welding processes play a paramount role in the expanding application of Al and its alloys, and have been considerably investigated in recent years. Tungsten inert gas (TIG) welding, as one of the most prevalent welding methods, has been widely used for welding aluminum alloys because of its advantages of utility and economy. In TIG welding, there is an electric arc between the base metals and a non-consumable tungsten electrode, which is used for melting and joining filler metals by heating them. The chemical composition of filler metal has a considerable impact on the microstructures and mechanical properties of the TIG weld joints [2,3].

Recently, aluminum matrix composites (AMCs) have become one of the most promising materials owing to their outstanding properties, e.g., low thermal expansion and good wear resistance [4]. Therefore, a number of researchers have attempted to create a novel fabrication of the composite filler metals with prospective mechanical properties by introducing ceramic particles to the filler metals in the past few years. M. Fattahi et al. [5,6,7,8] added ceramic particles into the filler metals using the accumulative roll bonding (ARB) approach, which revealed that the ceramic particles refined the α-Al grains and the mechanical properties of the TIG welds were improved. Maximilian Sokoluk et al. [9] applied salt-assisted nanoparticle incorporation and a hot extrusion process to produce composite filler metals. Results showed that the TiC particles could modify the second phases of the welds and eliminate the hot crack susceptibility of the welds. However, most researchers have focused on the mechanical properties of the welds, and the corrosion behavior of the welds has not drawn a lot of attention.

It has been reported that the corresponding properties of AMCs are related to the method of the fabrication being adopted [10,11,12,13]. In the liquid phase process, the ceramic particles generally are not wetted well with the liquid metal matrix, and because of their density corresponding to the liquid metal, the particles are apt to sink or float. Although solid-state method or powder metallurgy can easily produce metal matrix composites (MMCs) with improved mechanical properties, it is difficult to apply these techniques in industrial applications owing to their large cost [14,15]. Therefore, semi-solid casting is the most promising technology for application in large scale production for its low cost of processing, simplicity and the wide selection of particles [16,17,18].

Accordingly, the present work aims to combine the semi-solid stir casting procedure with the multi-pass rolling process to fabricate TiC_p_/Al-5Mg composite weld wires. The 5083-H116 aluminum alloys were joined by TIG welding using TiC_p_/Al-5Mg composite weld wires. The influence of micro TiC particles on the mechanical properties and corrosion behaviors of the weld joints were investigated.

## 2. Experimental Procedures

### 2.1. Fabrication of the Composite Filler Metals

**Figure 1:**
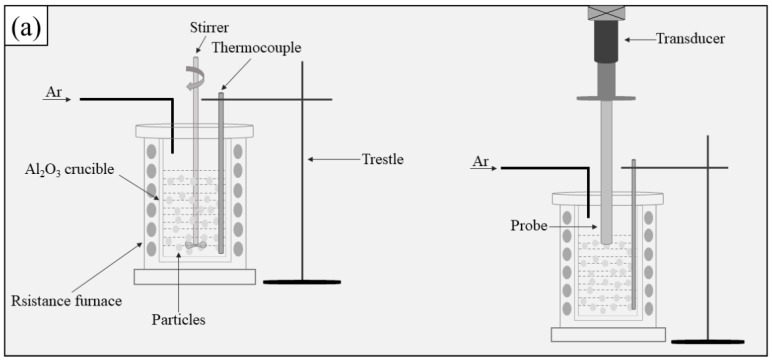

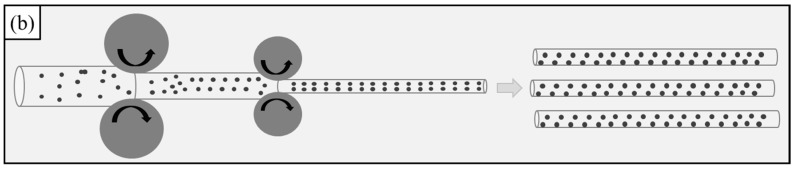
Schematic illustration of the fabrication of TiC_p_/Al-5Mg composite fillers: (**a**) Al-5Mg alloy and its composites preparation; (**b**) multi-pass rolling.

**Figure 2:**
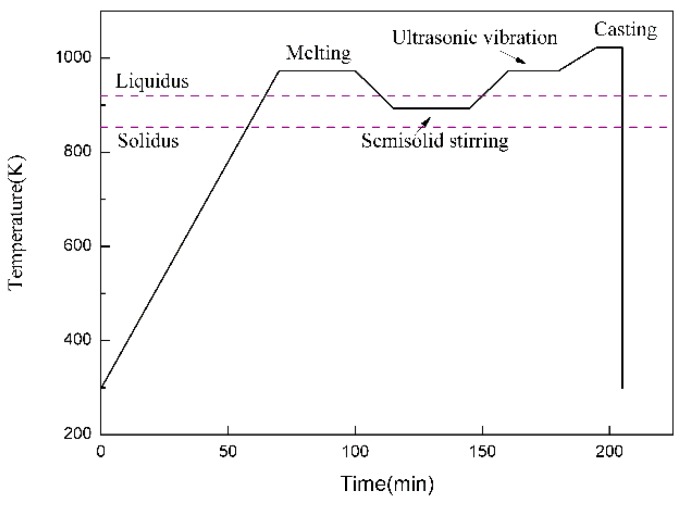
The diagram indicating the temperature–time relationship during casting.

Commercially pure aluminum (99.97%) and magnesium, and TiC ceramic particles with diameters of 2–4 μm as reinforcement were selected to produce Al-5Mg alloy and its composites. Figure 1 exhibits the manufacturing processes of the composite filler metals and Figure 2 shows the temperature–time diagram during the casting. The commercially pure aluminum and magnesium were melted at 973 K in an Al_2_O_3_ crucible. Then, the TiC particles, which were preheated to 473 K to remove moisture and gas from the surface of the particles, were quickly introduced into the melt; the melt was stirred when the temperature was cooled to 893 K, and the melt turned to a semi-solid state. After stirring evenly, the stirrer was removed from the melt. Then, the melt was again elevated to 973 K and subjected to ultrasonic processing for 15 min. After ultrasonic processing, the melt was heated to 1023 K for pouring into a preheated steel mold. For comparison, Al-5Mg alloy was also prepared as a reference material. The cast ingots were machined into 6 rods with dimension of Φ 5 mm × 150 mm, and then the rods were homogenized at 723 K for 15 h. Finally, the homogenized rods were processed into fine filler wires by multi-pass rolling (Figure 1b).

### 2.2. Welding Procedure

In the present study, 4-mm thick sheets of 5083-H116 aluminum alloy were selected as the base metal. The sheets were machined into a dimension of 150 mm × 75 mm × 3 mm with an I-groove. The welding joints were fabricated by TIG welding with the Al-5Mg and its composite filler wires. In the TIG welding processes, the arc current, voltage, flow rate of the argon gas and applied heat input were 140 A, 25 V, 14 L/min and 11.7 J/mm, respectively.

### 2.3. Microstructure Characterization

Specimens for metallographic investigation were cut from the weld joints, then prepared using standard polishing techniques before etching using Keller’s reagent or anodical oxidation (“Barker etch”). Microstructure of the weld joints were captured by a Leica DMi8 optical microscope (Leica, Weztlar, Germany) and the average grain size was calculated by the linear intercept method. The distribution of TiC particles and the intermetallic phases of the weld joints were evaluated on a Hitachi S-4800 scanning electron microscope (SEM, Hitachi, Tokyo, Japan) equipped with an energy dispersive spectrometer (EDS), and the applied spot size and accelerated voltage for EDS analysis were 1 μm^3^ and 15 keV, respectively.

### 2.4. Mechanical Properties Test and Corrosion Test

To evaluate the mechanical properties of the welds, the transverse tensile specimens were machined perpendicular to the TIG weld direction and the dimensions were 60 mm of gauge length and 8 mm of gauge width, respectively. Room temperature tensile tests were carried out at the loading speed of 1 mm/min and three samples were tested for the various conditions. The fractography of specimens were observed on a Hitachi S-4800 scanning electron microscope. Vickers microhardness with a load of 200 g for 15 s was tested on the cross-section perpendicular to the welding direction along the mid-thickness of the sheets using a VH-5 microhardness tester (Everone, Shanghai, China).

The corrosion behavior of the specimen in 3.5% NaCl aqueous solution was characterized through electrochemical polarization measurement. The specimens were machined to the dimension of 10 × 10 × 2 mm^3^ from the weld zone of the joints. A copper wire was connected to the back of each sample prior to embedding in an epoxy resin. The working area exposed to the solution was 1 cm^2^. Then, the specimen surfaces were abraded on 1500 silicon carbide paper and washed with distilled water. The reference electrode was a saturated calomel electrode (SCE) and the counter electrode was a platinum sheet. The Tafel curves were recorded at a sweep rate of 0.001 V/s. The corrosion current density (icorr) was evaluated using the Tafel extrapolation method.

In order to evaluate the stress corrosion cracking (SCC) resistance of the welds, slow strain rate testing (SSRT) was employed. The tensile test specimen with a gauge length of 20 mm, a width of 4.5 mm and a thickness of 3 mm was prepared perpendicular to the welding direction. During the test, the gauge length of the samples was completely immersed in a 3.5% NaCl aqueous solution. Tensile tests were performed on a WDML-10 testing machine at room temperature with a strain rate of 2 × 10^−6^ s^−1^.

## 3. Results and Discussion

### 3.1. Microstructure

**Figure 3:**
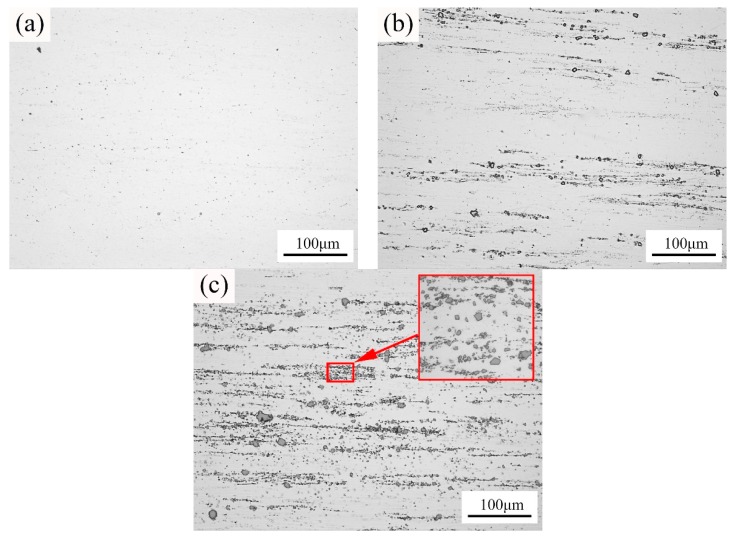
Optical microscopy micrographs of the Al-5Mg filler wires containing: (**a**) 0 wt% TiC particles; (**b**) 2 wt% TiC particles; (**c**) 4 wt% TiC particles.

Figure 3 exhibits the optical micrographs of the composite filler wires containing different contents of TiC particles produced by semi-solid stir casting and the multi-pass rolling method. It is obvious that the reinforced particles were successfully introduced into the basic alloy through the semi-solid stir casting process. In Figure 3c, the micrograph of the 4 wt% TiCp/Al-5Mg composites shows that a homogeneous distribution of TiC particles in the aluminum matrix is obtained after multi-pass rolling. Moreover, there are no evident defects (such as porosity) in the composites. Therefore, the present work represents an efficient method to fabricate TiCp/Al-5Mg composites weld wires with homogeneous dispersion of the TiC particles in the aluminum matrix.

**Figure 4:**
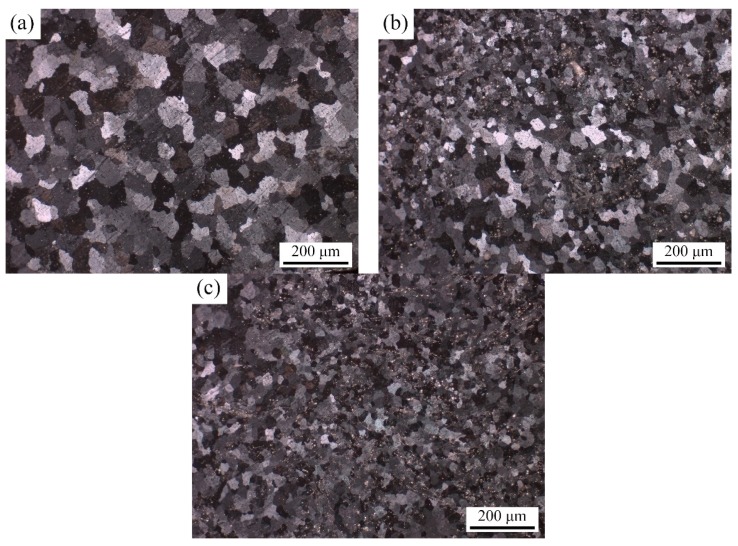
Optical micrographs under polarized light condition of weld joints with composite filler wires containing: (**a**) 0 wt% TiC particles; (**b**) 2 wt% TiC particles; (**c**) 4 wt% TiC particles.

Figure 4 reveals the optical micrographs under polarized light condition of weld joints with composite filler wires containing different contents of TiC particles. It is evident that the α-Al grain size was considerably refined in the weld joints with the addition of TiC particles to the filler wires. The average grain size of α-Al in the weld prepared with Al-5Mg filler and 2 and 4 wt% TiCp/Al-5Mg composite filler wires were calculated as 87.14 μm, 51.55 μm and 27.54 μm, respectively. As a result of the heterogeneous nucleation by TiC particles, the grains in the welds prepared with TiCp/Al-5Mg composite filler wires were gradually refined. The small lattice mismatch between the aluminum matrix and TiC particles leads to the decrease in the activation energy for nucleation and thus enhances the potency of heterogeneous nucleation sites [19]. Therefore, the refinement of α-Al grains could be attributed to the capability of TiC particles to nucleate α-Al grains and restricted growth of α-Al grains during solidification.

**Figure 5:**
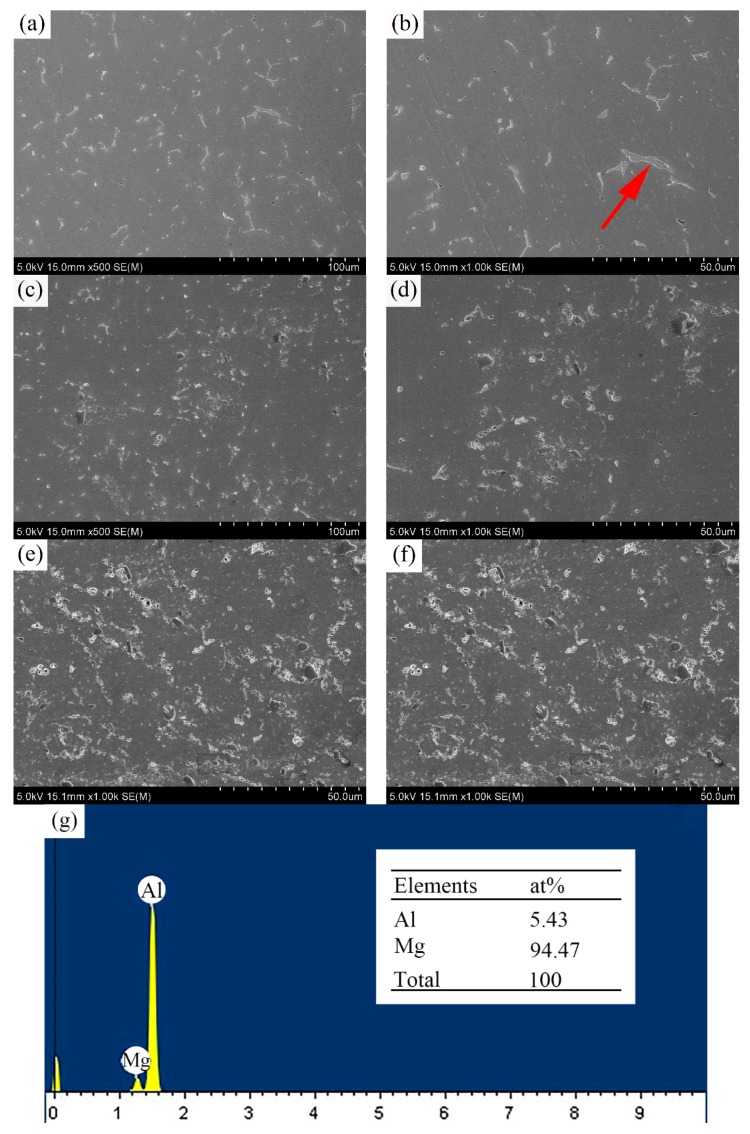
SEM images of weld joints with composites fillers containing (**a**) and (**b**) 0 wt% TiC particles; (**d**) and (**c**) 2 wt% TiC particles; (**e**) and (**f**) 4 wt% TiC particles and (**g**) corresponding EDS point analysis taken from the arrowed second phases in (**b**).

Figure 5 illustrates the SEM images of weld joints with composite fillers containing different contents of TiC particles. As shown in Figure 5a, coarse and continuous second phases were observed in the weld without TiC particles. According to the EDS analysis in Figure 5g, the arrowed phases mainly contained Al and Mg elements, which can be regarded as Mg_5_Al_8_ phases (β phases), whereas it is observed that the formation of Al-Mg eutectic phases was inhabited as the contents of TiC particles were introduced into the weld joints [20]. In Figure 5e, it is obvious that a number of TiC particles were distributed inside the grains while the majority of TiC particles were agglomerated on the grain boundaries, and no coarse and continuous β phases were found in the weld joints containing 4 wt% TiC particles. During the TIG welding process, the TiC particles were “captured” by α-Al grains owing to the fast solidification rate of the weld couples and, as a result, the relative TiC particles uniformly distributed in the grain. On the other hand, because of the poor wettability, some TiC particles were “pushed” to the grain boundaries [7]. Consequently, the relative TiC particles tend to form the agglomerates in the grain boundaries. Moreover, the existence of TiC particles limits the diffusion and migration of Mg elements to grain boundaries, and inhibits the formation and growth of the β phases.

### 3.2. Mechanical Properties

**Figure 6:**
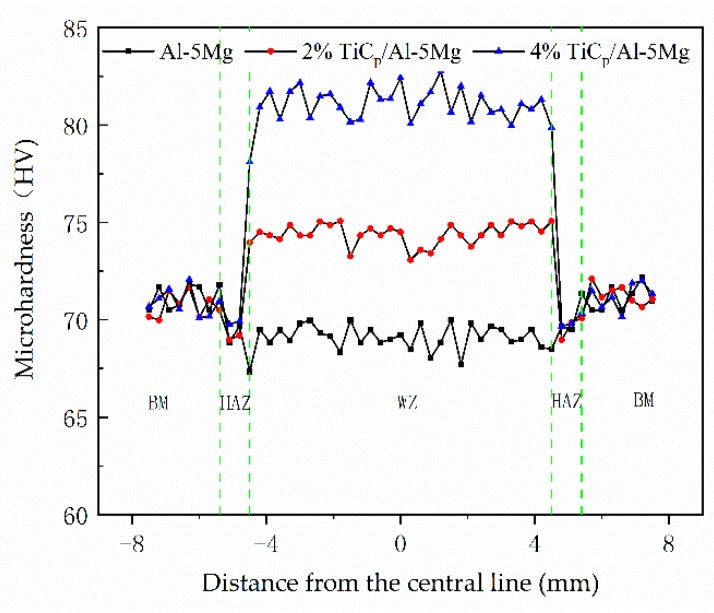
Microhardness distributions of the weld joints produced with different contents of TiC particles.

The Vickers microhardness distribution along the transverse cross-section of the joints with composite filler wires containing different contents of TiC particles are shown in Figure 6. The result shows that the highest microhardness value is in the weld zone and this dropped suddenly in the heat affected zone when the joints were prepared with filler wire containing TiC particles. However, the microhardness values of the weld zone are lower than that of heat affected zone in the joints prepared with filler wire without TiC particles. It was obvious that the hardness values in the weld region of the joints increases as the addition of TiC particles of the weld filler wires increases.

**Figure 7:**
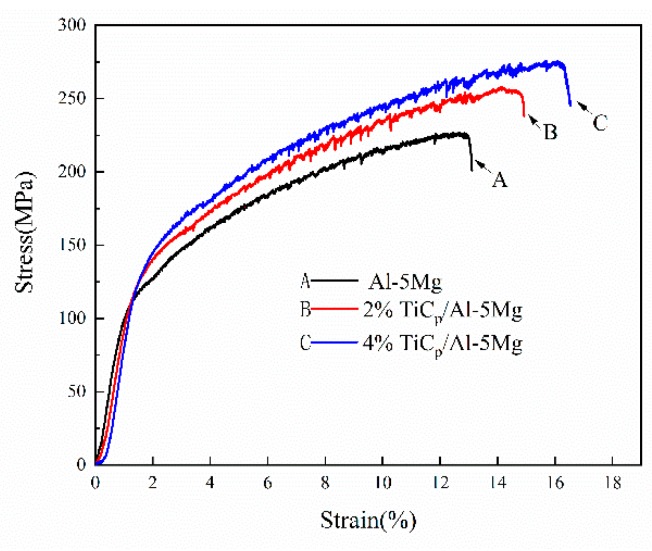
Engineering stress–strain curves of the welds.

Figure 7 shows the tensile properties of the weld joints with composite filler wires containing 0, 2, and 4 wt% TiC particles at room temperature. It can be seen that the ultimate tensile strength and ductility of the reinforced welds are considerably increased compared with the reference weld. The ultimate tensile strength (UTS) of the weld joints prepared with filler wires containing 4 wt% TiC particles is 276.02 MPa, whereas the value of the unreinforced weld is only 226.71 MPa. The ductility of the reinforced weld joints also increased. The weld joints prepared with filler wires containing 4 wt TiC particles exhibited considerably higher elongation of about 20.7% in comparison with the unreinforced weld having 13.1%.

The simultaneous improvement in tensile strength and elongation in the reinforced welds can be also attributed to the changes in the microstructure of the weld joints. It is well known that the shape, size and distribution of the second phase and the grain size of the matrix phase have a significant influence on the mechanical properties of alloys [21,22]. In this study, the strength and ductility mechanisms can be ascribed to the following factors: (i) the introduction of hard TiC ceramic particles into the welds increases the dislocation density [23]; (ii) the volume fraction of grain boundaries increases in the weld produced with TiC particles (Figure 4); and (iii) the β phases refined or dissolved in the Al matrix in the weld joints (Figure 5). Generally speaking, the volume fraction of grain boundaries increases with decreasing grain size and hence the localized plastic deformation is restrained by the grain boundaries. It is well known that the yield strength of metals increases with the refinement of grains according to the Hall–Petch relationship [24]:

∆δ_y_ = K_y_D ^(−1/2)^ (1)

where ∆δ_y_ is the yield strength, K_y_ is the Hall–Petch constant and D is the average grain size. At the same volume fraction, the smaller the particle size, the smaller the particle spacing and hence the more obvious the strengthening effect [21]. As can be seen in Figure 4 and Figure 5, introducing TiC particles into the weld joints has a sufficient effect on refining the grain of the Al matrix and β phases during TIG welding, for the capability of TiC particles to nucleate α-Al grains. Therefore, the microhardness and UTS improves in the welds prepared with TiCp/Al-5Mg composite fillers. Furthermore, thermal strains tend to form around TiC particles because of the difference in thermal expansion coefficients of TiC particles and the aluminum matrix, which also increases the microhardness value and UTS of the weld [25,26].

**Figure 8:**
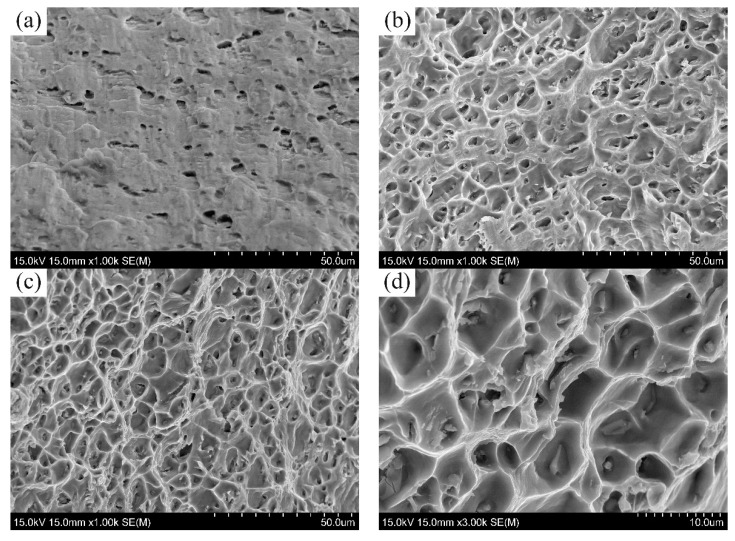
SEM images of the fractography of 5083 weld joints prepared with: (**a**) Al-5Mg, (**b**) 2 wt% TiC_p_/Al-5Mg, (**c**) 4 wt% TiC_p_/Al-5Mg and (**d**) higher magnification image of (**c**).

The fractographies of 5083 weld joints containing different contents of TiC particles were analyzed by SEM. As is shown in Figure 8, the fractography of the unreinforced weld joint was covered by a few large dimples and extensive distribution of facets, which indicate the brittle fracture mode owing to the large grains in the α-Al matrix including coarse and continuous β phases (Figure 4a and Figure 5a), while the fractographies of the weld prepared with TiCp/Al-5Mg composite filler wires are dominated with dimples and micro-voids, which indicate a typical ductile fracture (Figure 8b–d). The fractography of welds prepared with 4 wt% TiCp/Al-5Mg composite filler wires consists of finer and more homogeneous dimples compared with the weld joints containing 2 wt% TiC particles, which is beneficial to the uniform elongation. Moreover, M. Fattahi et al. reported that there is a corresponding relationship between the decrease in the dimple size and increase in the strength [7]. The higher magnification image of the fractography of the weld prepared with 4 wt% TiC/Al-5Mg composite filler wires clearly shows that TiC particles were well distributed in the aluminum matrix, which reveals a good interfacial bonding between the basic metals and TiC particles (Figure 8d).

### 3.3. Corrosion Behavior

**Figure 9:**
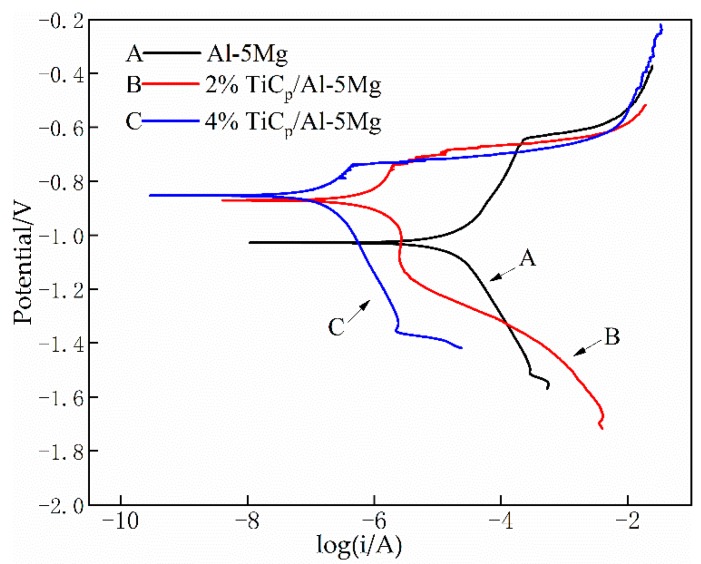
Polarization curves of the weld with and without TiC particles.

Figure 9 presents the potentiodynamic polarization plots of 5083 weld joints with and without TiC reinforced particles; their kinetic parameters are listed in Table 1. It was noticeable that the TiC reinforced particles have an influence on the polarization characteristic, for the variation in the corrosion potential (E_corr_), the corrosion current density (I_corr_) and the reaction kinetics. The weld joints with TiC reinforced particles have lower I_corr_ values. The weld joint without reinforced particles has a high rate of anodic kinetics because of the high I_corr_ values. In the weld prepared with TiCp/Al-5Mg composite filler wires, the corrosion potential moved towards a positive value, showing passive behavior. With the contents of TiC particles in the weld joints increasing, the corrosion potential shifts to a higher state.

As shown in Table 1, the I_corr_ values of the weld without TiC reinforced particles are much higher than the I_corr_ measured for the weld prepared with TiC composite filler wires, and the I_corr_ value was reduced further with increasing the contents of TiC particles. TiC particles are basically inert in the corrosive solution, which would not accelerate the local galvanic effect. The uniformly distributed TiC particles reduced the surface area of the metal exposed to the solution, thus reducing the corrosion rate. On the other hand, it is well known that β-phases act as anodic during the potentiodynamic polarization testing because of the high Mg contents [27]. As shown in Figure 5, the continuous and large β-phases were obtained in weld joints without TiC particles. However, the form of β-phases was restricted when the particles were introduced into the weld joints, which indicates that adding TiC particles into the weld wires can improve the corrosion resistance of the weld joints. A number of researchers have also reported that intermetallic compounds have a negative influence on the corrosion rate in aluminum alloys and aluminum matrix composites, in accord with the present work [28,29,30].

**Figure 10:**
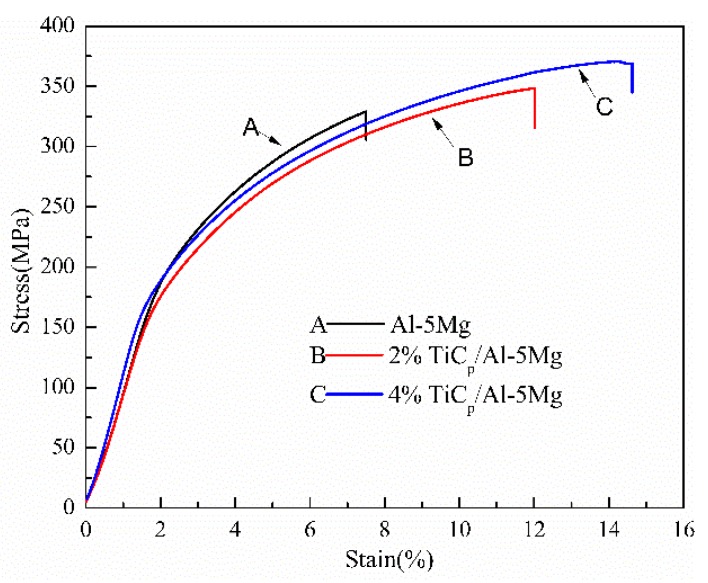
Stress–strain curves of the weld with and without TiC particles during slow strain rate testing (SSRT) in 3.5 wt% NaCl solution.

Slow strain rate testing (SSRT) was employed to investigate stress corrosion cracking of the welds. Figure 10 shows stress–strain curves of the welds prepared with Al-5Mg, 2 wt% TiCp/Al-5Mg and 4 wt% TiCp/Al-5Mg filler metals during the SSRT in 3.5 wt% NaCl solution. The ultimate tensile strength (UTS) and the time to failure of the welds containing different content of TiC particles during SSRT are summarized in Table 2. It is obvious that the tensile strength and time to failure of unreinforced welds are low. With the increase of the TiC particle content in the weld joints, the tensile strength and time to failure increase. This is the result of the refinement or dissolution of β-phases in the Al matrix in the weld joints. The formation of the β-phase has a tendency to provide a path for crack propagation, and this will contribute to the increase of the susceptibility to stress corrosion cracking [29,31,32]. In the welds without reinforcement, the coarse β-phases distribute continuously along the grain boundaries. The grain boundaries are inclined to dissolve away completely after the dissolution of the coarse and continuous β-phases during the SSRT process. Although the formation of β-phases was restricted in the reinforcement welds, the β-phase varied from coarse and continuous to discontinuous and thin, or even disappeared, in the grain boundaries. Therefore, the grain boundaries were only partially dissolved in the reinforcement welds. As a result, the SCC susceptibility of the welds decreased as micro-TiC particles were introduced into the weld.

### 3.4. Al/TiC Interface Calculation

To evaluate the corrosion mechanism, the electron work function (EWF) and the electronic structure of the composite were characterized by first principle calculation based on density functional theory. EWF, the energy required to withdraw an electron completely from a surface, is a key physical property involved in the evaluation of corrosion resistance. It is calculated as the difference between the vacuum level E_vacuum_ and the Fermi level E_Fermi_:

Φ = E_vacuum_ − E_Fermi_

A surface with high EWF is expected to have good corrosion resistance. Table 3 shows the lattice constants and electron work functions (EWF) of Al and TiC. Compared with other theoretical and experimental values, our result is reasonable. The calculated EWF of Al ranges from 3.87 eV for the Al (111) surface to 4.14 eV for the Al (100) surface, agreeing with the experimental value of polycrystalline Al, 4.28 eV [33]. For TiC, the calculated lattice constant, 4.33 Å, is consistent with other density functional theory (DFT) results and the experiment. Moreover, the lowest EWF of a TiC surface is of (111) with Ti termination, with a value of 4.59 eV, which is still larger than 4.14 eV, the highest EWF of an Al surface. Although the TiC surfaces have higher EWF than the Al surfaces, the electrochemical potential depends on the overall work function [34]. In general, adding an element having a higher work function to a host metal with a lower work function may elevate overall work function [35], due to the increase in the valence or free electron density [36]. With regard to the introduction of ceramic particles, the conclusion is still correct, and many experimental results have shown that the overall work function increases with the incorporation of ceramic composite particles [34,37]. However, the exact mechanism for the increase in overall work function caused by the particles remains unclear.

**Figure 11:**
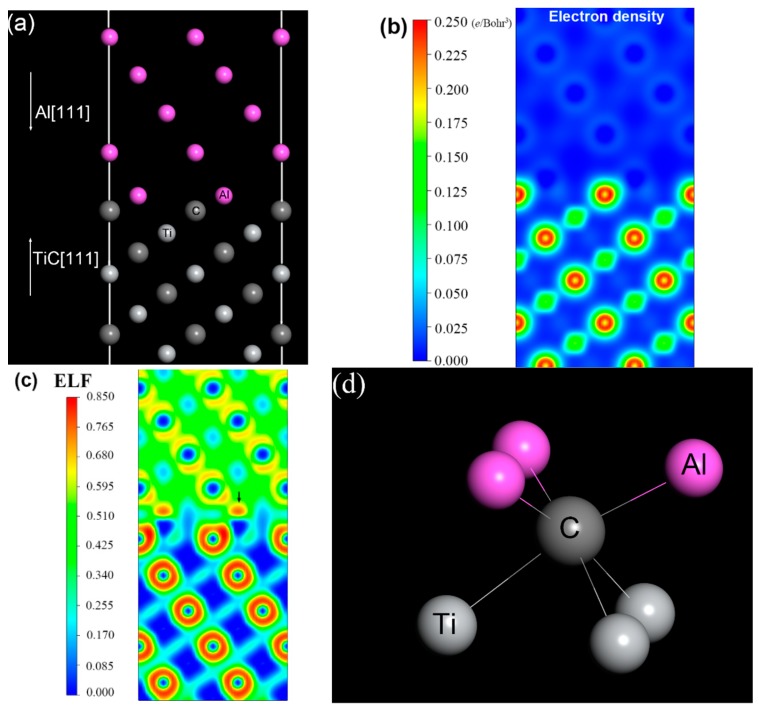
(**a**) The atomic configuration at the Al–TiC interface; (**b**) the distribution of valence electron density; (**c**) electron location function (ELF) of valence electrons; (**d**) the model of Al–TiC.

To address the issue, an interfacial model of TiC/Al was built in order to investigate the valence electrons near the interface. The supercell of TiC/Al, in which a nine-layer slab of Al (111) plane is connected with an eight-layer slab of C-terminated TiC (111) plane, is exhibited in Figure 11a. This configuration has maximal adhesion energy according to the result reported by Sun et al. [38]. The calculations were performed in the framework of DFT with the projected augmented wave (PAW) method [39], using the Vienna ab initio simulation package (VASP) [40], which is effective in describing the electronic structure of interfaces. Electron exchange and correlation were described by the Perdew–Burke–Ernzerhof (PBE) generalized gradient approximation (GGA) [41]. Γ-point centered 7 × 7 × 1 and 9 × 9 × 1 k-point meshes were used for atomic relaxation and electronic structure analysis, respectively. The plane–wave energy cutoff was set to 500 eV. The convergence in energy and force was set to 10^−5^ eV and 10^−3^ eV/Å, respectively. The calculated electron density distribution and electron location function (ELF) are presented in Figure 11b,c, respectively. It can be found that the electron density of C atoms at the interface shifts to the Al layer, based on the analysis of electron density distribution. ELF measures the possibility of finding an electron in the neighborhood space and provides a method for the mapping of electron pair probability [42]. The red region in Figure 11c suggests that a considerable charge accumulation occurs at the interfacial Al atoms, as indicated by the arrow. The result indicates that C–Al bonds are formed at the interface. Here, each C atom bonds with three Al atoms simultaneously, as shown in Figure 11d.

**Figure 12:**
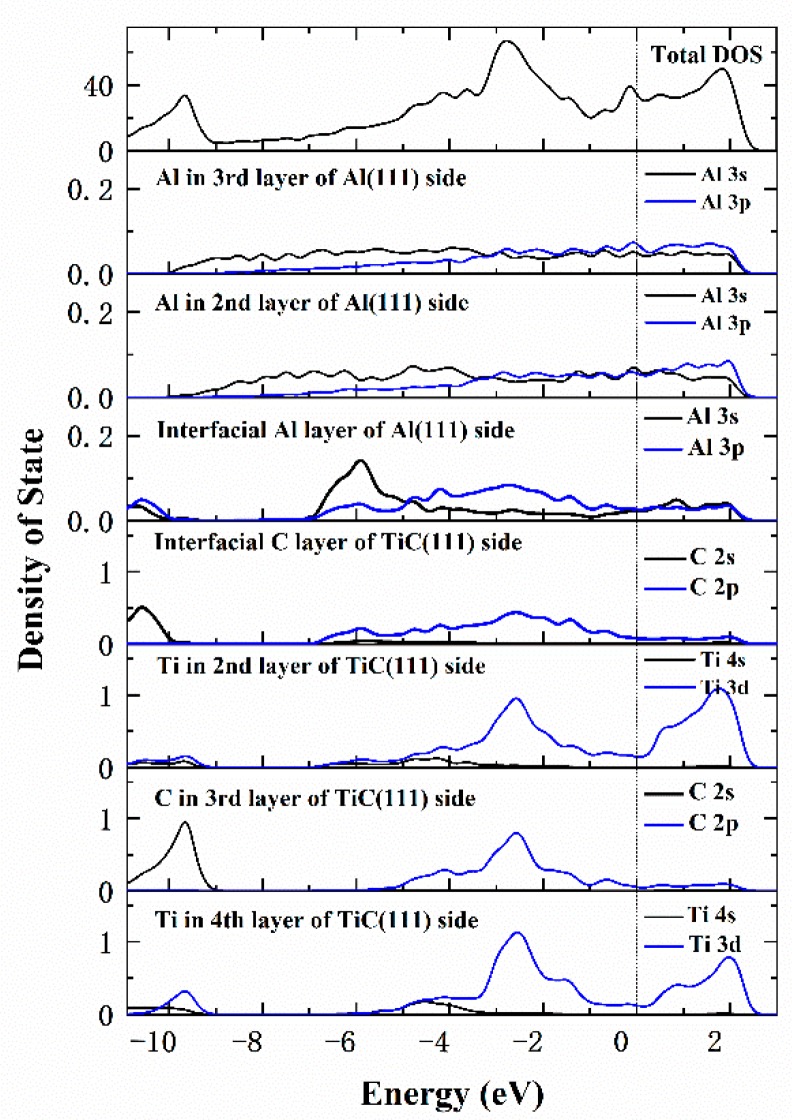
Total and partial density of states of interfacial atoms. The vertical dashed line indicates the Fermi level.

For a further insight into the electron distribution and the bonding behavior, the density of state (DOS) is investigated, as illustrated in Figure 12. Obviously, the DOS of interfacial Al atoms is different from the Al atoms in bulk. There are two local states in the vicinity of −6.0 eV, which exactly overlap with the electronic distribution of interfacial C atoms. These are the hybridization between Al-3s and C-2p orbits, and indicate that the Al–C bonds have covalent features. The formation of the covalent bonds inevitably induces the transfer of valence electrons from C atoms, and consequently an increase in electron density. Consequently, the overall work function is raised. The electrochemical potential is proportional to the work function of an alloy, and the contact potential difference at the alloy/solution interface is often a constant for a certain solution [34,43]. Therefore, the corrosion potential increases with the fraction of TiC in the composite, which is consistent with previous experimental observation. Moreover, the Al–C covalent bonds can inhibit the migration of free electrons through the interface, and result in a higher corrosion resistance, which is also responsible for the decrease of the corrosion current density of the reinforced weld.

## 4. Conclusions

TiCp/Al-5Mg composite filler wires with various contents of TiC particles were successfully fabricated by a semi-solid stir casting mixed multi-pass rolling process. The 5083-H116 aluminum alloys were joined by TIG welding using TiCp/Al-5Mg composite weld wires. The microstructure, corresponding mechanical properties and the corrosion behavior of welds were investigated.

(1) By applying a semi-solid stir casting and multi-pass rolling process, TiC particles were homogeneously distributed in the composite fillers. As a result, the welds prepared with TiCp/Al-5Mg composite wires exhibited a homogeneous dispersion of TiC particles. The introduction of TiC particles to the welds played an effective role in decreasing the grain size of the primary α-Al because of the increased amount of heterogeneous nucleation sites and the retarded grain growth. In comparison with the unreinforced weld sample, the microhardness and tensile strength of the reinforced weld specimens were considerably improved.
(2) The reinforced weld samples also exhibited better corrosion resistance compared with the unreinforced weld in 3.5 wt% NaCl solution. The SSRT result reveals that the reinforced welds had a better SCC resistance than that of the unreinforced weld.
(3) The first-principle calculation revealed that the improvement of the reinforced weld in corrosion resistance owed to the electron localization in the interfacial region between the Al matrix and TiC particles.

Hence, it can be inferred that the TiCp/Al-5Mg composite wires can be applied as novel fillers for TIG welding of aluminum and its alloys.

## Figures and Tables

**Table 1:** Electrochemical parameters from polarization curves in Figure 9.

Particle Contents (%)	E_corr_ (V/SCE)	I_corr_ (μA·cm^−2^)	r (mm/year)
0	−1.0295	15.2210	0.3673
2	−0.8749	4.8559	0.1171
4	−0.8574	2.0699	0.0500

**Table 2:** The ultimate tensile strength (UTS) and time to failure of the welds.

Samples	UTS(MPa)	Time to Failure (h)
Al-5Mg	329.0	31.2
2%TiCp/Al-5Mg	348.3	50
4%TiCp/Al-5Mg	370.5	60.7

**Table 3:** Lattice constants and electron work functions of Al and TiC.

			This Work	Other Theoretical Results	Experimental Values
Al	Lattice constant (Å)		4.04	4.05 [38]	4.05 [44,45]
	EWF (eV)	(100)	4.14	4.35 [46]	4.41 ± 0.02 [47,48]
		(110)	3.95	3.97 [46]	4.28 ± 0.02 [44,45]
		(111)	3.87	4.16 [46]	4.24 ± 0.03 [44,45]
TiC	Lattice constant (Å)		4.33	4.33 [49]	4.33 [50]
	EWF (eV)	(100) with Ti and C termination	5.23	4.94 [51]	4.1 [52]
		(111) with Ti termination	4.59	-	-
		(111) with C termination	5.11	-	-

## References

[1] Kelly J.C., Sullivan J.L., Burnham A., Elgowainy A. (2015). Impacts of vehicle weight reduction via material substitution on life-cycle greenhouse gas emissions. Environ. Sci. Technol..

[2] Fattahi M., Nabhani N., Rashidkhani E., Fattahi Y., Akhavan S., Arabian N. (2013). A new technique for the strengthening of aluminum tungsten inert gas weld metals: Using carbon nanotube/aluminum composite as a filler metal. Micron.

[3] Fattahi M., Gholami A.R., Eynalvandpour A., Ahmadi E., Fattahi Y., Akhavan S. (2014). Improved microstructure and mechanical properties in gas tungsten arc welded aluminum joints by using graphene nanosheets/aluminum composite filler wires. Micron.

[4] Beigi Khosroshahi N., Taherzadeh Mousavian R., Azari Khosroshahi R., Brabazon D. (2015). Mechanical properties of rolled a356 based composites reinforced by cu-coated bimodal ceramic particles. Mater. Des..

[5] Ahmadi E., Ranjkesh M., Mansoori E., Fattahi M., Mojallal R.Y., Amirkhanlou S. (2017). Microstructure and mechanical properties of Al/ZrC/TiC hybrid nanocomposite filler metals of tungsten inert gas welding fabricated by accumulative roll bonding. J. Manuf. Sci. Process.

[6] Ramkumar K.R., Natarajan S. (2018). Investigations on microstructure and mechanical properties of TiO_2_ nanoparticles addition in Al 3003 alloy joints by gas tungsten arc welding. Mater. Sci. Eng. A.

[7] Fattahi M., Mohammady M., Sajjadi N., Honarmand M., Fattahi Y., Akhavan S. (2015). Effect of TiC nanoparticles on the microstructure and mechanical properties of gas tungsten arc welded aluminum joints. J. Mater. Process. Technol..

[8] Dabiri A.R., Yousefi Mojallal R., Ahmadi E., Fattahi M., Amirkhanlou S., Fattahi Y. (2015). Effect of ZrO_2_ nanoparticles on the impact properties of shielded metal arc welds. Mater. Lett..

[9] Sokoluk M., Cao C., Pan S., Li X. (2019). Nanoparticle-enabled phase control for arc welding of unweldable aluminum alloy 7075. Nat. Commun..

[10] Radi Y., Mahmudi R. (2010). Effect of Al_2_O_3_ nano-particles on the microstructural stability of AZ31 Mg alloy after equal channel angular pressing. Mater. Sci. Eng. A.

[11] Yang Y., Lan J., Li X. (2004). Study on bulk aluminum matrix nano-composite fabricated by ultrasonic dispersion of nano-sized sic particles in molten aluminum alloy. Mater. Sci. Eng. A.

[12] Wang H.Y., Jiang Q.C., Zhao Y.Q., Zhao F., Ma B.X., Wang Y. (2004). Fabrication of TiB_2_ and TiB_2_–TiC particulates reinforced magnesium matrix composites. Mater. Sci. Eng. A.

[13] Razavi Tousi S.S., Yazdani Rad R., Salahi E., Mobasherpour I., Razavi M. (2009). Production of Al–20 wt.% Al_2_O_3_ composite powder using high energy milling. Powder Technol..

[14] Razavi-Tousi S.S., Yazdani-Rad R., Manafi S.A. (2011). Effect of volume fraction and particle size of alumina reinforcement on compaction and densification behavior of Al–Al_2_O_3_ nanocomposites. Mater. Sci. Eng. A.

[15] Razavi Hesabi Z., Hafizpour H.R., Simchi A. (2007). An investigation on the compressibility of aluminum/nano-alumina composite powder prepared by blending and mechanical milling. Mater. Sci. Eng. A.

[16] Vieira A.C., Sequeira P.D., Gomes J.R., Rocha L.A. (2009). Dry sliding wear of Al alloy/sicp functionally graded composites: Influence of processing conditions. Wear.

[17] Rajan T.P.D., Pillai R.M., Pai B.C. (2010). Characterization of centrifugal cast functionally graded aluminum-silicon carbide metal matrix composites. Mater. Charact..

[18] Zhang L.-J., Yang D.-L., Qiu F., Wang J.-G., Jiang Q.-C. (2015). Effects of reinforcement surface modification on the microstructures and tensile properties of SiCp/Al2014 composites. Mater. Sci. Eng. A.

[19] Dudiy S.V., Lundqvist B.I. (2004). Wetting of TiC and TiN by metals. Phys. Rev. B.

[20] Nie K.B., Wang X.J., Wu K., Hu X.S., Zheng M.Y., Xu L. (2011). Microstructure and tensile properties of micro-SiC particles reinforced magnesium matrix composites produced by semisolid stirring assisted ultrasonic vibration. Mater. Sci. Eng. A.

[21] Yang H., Gao T., Wu Y., Zhang H., Nie J., Liu X. (2018). Microstructure and mechanical properties at both room and high temperature of in-situ TiC reinforced Al–4.5Cu matrix nanocomposite. J. Alloys Compd..

[22] Chao S., Min S., Wang Z., He Y. (2011). Effect of particle size on the microstructures and mechanical properties of SiC-reinforced pure aluminum composites. J. Mater. Eng. Perform..

[23] Habibnejad-Korayem M., Mahmudi R., Poole W.J. (2009). Enhanced properties of Mg-based nano-composites reinforced with Al2O3 nano-particles. Mater. Sci. Eng. A.

[24] Sheikhi M., Malek Ghaini F., Assadi H. (2015). Prediction of solidification cracking in pulsed laser welding of 2024 aluminum alloy. Acta Mater..

[25] Selvam J.D.R., Dinaharan I., Philip S.V., Mashinini P.M. (2018). Microstructure and mechanical characterization of in situ synthesized AA6061/(TiB_2_+Al_2_O_3_) hybrid aluminum matrix composites. J. Alloys Compd..

[26] Muralidharan N., Chockalingam K., Dinaharan I., Kalaiselvan K. (2018). Microstructure and mechanical behavior of AA2024 aluminum matrix composites reinforced with in situ synthesized ZrB_2_ particles. J. Alloys Compd..

[27] Seong J., Frankel G.S., Sridhar N. (2016). Inhibition of stress corrosion cracking of sensitized AA5083. Corrosion.

[28] Aballe A., Bethencourt M., Botana F.J., Cano M.J., Marcos M. (2003). Influence of the cathodic intermetallics distribution on the reproducibility of the electrochemical measurements on AA5083 alloy in nacl solutions. Corros. Sci..

[29] Goswami R., Spanos G., Pao P.S., Holtz R.L. (2010). Microstructural evolution and stress corrosion cracking behavior of Al-5083. Metall. Mater. Trans. A.

[30] Searles J.L., Gouma P.I., Buchheit R.G. (2001). Stress corrosion cracking of sensitized AA5083 (Al-4.5Mg-1.0Mn). Metall. Mater. Trans. A.

[31] Li H., Zhang X., Chen M., Li Y., Liang X. (2007). Effect of pre-deformation on the stress corrosion cracking susceptibility of aluminum alloy 2519. Rare Met..

[32] Han M.-S. (2008). Optimization of corrosion protection potential for stress corrosion cracking and hydrogen embrittlement of 5083-H112 alloy in seawater. Met. Mater. Int..

[33] Eastment R.M., Mee C.H.B. (1973). Work function measurements on (100), (110) and (111) surfaces of aluminium. J. Phys. F Met. Phys..

[34] Mosleh-Shirazi S., Hua G., Akhlaghi F., Yan X., Li D. (2015). Interfacial valence electron localization and the corrosion resistance of Al-SiC nanocomposite. Sci. Rep..

[35] Lu H., Hua G., Li D. (2013). Dependence of the mechanical behavior of alloys on their electron work function—An alternative parameter for materials design. Appl. Phys. Lett..

[36] Hua G., Li D. (2011). Generic relation between the electron work function and young’s modulus of metals. Appl. Phys. Lett..

[37] Kiourtsidis G.E., Skolianos S.M., Pavlidou E.G. (1999). A study on pitting behaviour of AA2024/SiCp composites using the double cycle polarization technique. Corros. Sci..

[38] Sun T., Wu X., Wang R., Li W., Liu Q. (2017). First-principles study on the adhesive properties of Al/TiC interfaces: Revisited. Comput. Mater. Sci..

[39] Kresse G., Joubert D. (1999). From ultrasoft pseudopotentials to the projector augmented-wave method. Phys. Rev. B.

[40] Kresse G., Furthmüller J. (1996). Efficiency of ab-initio total energy calculations for metals and semiconductors using a plane-wave basis set. Comput. Mater. Sci..

[41] Perdew J.P., Wang Y. (1992). Accurate and simple analytic representation of the electron-gas correlation energy. Phys. Rev. B.

[42] Becke A.D., Edgecombe K.E. (1990). A simple measure of electron localization in atomic and molecular systems. J. Chem. Phys..

[43] Bockris J.O., Khan S. (1993). Surface Electrochemistry.

[44] Kamm G.N., Alers G.A. (1964). Low-temperature elastic moduli of aluminum. J. Appl. Phys..

[45] Syassen K., Holzapfel W. (1978). Isothermal compression of Al and Ag to 120 kbar. J. Appl. Phys..

[46] Jin Y., Liu M., Zhang C., Leygraf C., Wen L., Pan J. (2017). First-principle calculation of volta potential of intermetallic particles in aluminum alloys and practical implications. J. Electrochem. Soc..

[47] Fall C.J., Binggeli N., Baldereschi A. (1998). Anomaly in the anisotropy of the aluminum work function. Phys. Rev. B.

[48] Grepstad J.K., Gartland P.O., Slagsvold B.J. (1976). Anisotropic work function of clean and smooth low-index faces of aluminium. Surf. Sci..

[49] Liu L.M., Wang S.Q., Ye H.Q. (2004). Adhesion and bonding of the Al/TiC interface. Surf. Sci..

[50] Dunand A., Flack H.D., Yvon K. (1985). Bonding study of TiC and TiN. I. High-precision X-ray-diffraction determination of the valence-electron density distribution, Debye-Waller temperature factors, and atomic static displacements in TiC_0.94_ and TiN_0.99_. Phys. Rev. B.

[51] Hugosson H.W., Eriksson O., Jansson U., Ruban A.V., Souvatzis P., Abrikosov I.A. (2004). Surface energies and work functions of the transition metal carbides. Surf. Sci..

[52] He J.-W., Norton P.R. (1988). Fractional-order desorption of D2 from a Pd(110) surface. Surf. Sci..

